# A Reverse Transcription Recombinase-Aided Amplification Method for Rapid and Point-of-Care Detection of SARS-CoV-2, including Variants

**DOI:** 10.3390/v13091875

**Published:** 2021-09-19

**Authors:** Fengyun Li, Ping He, Dongyan Xiong, Yakun Lou, Qiaosheng Pu, Haixia Zhang, Huige Zhang, Junping Yu

**Affiliations:** 1 State Key Laboratory of Applied Organic Chemistry, Key Laboratory of Nonferrous Metals Chemistry and Resources Utilization of Gansu Province, Department of Chemistry, Lanzhou University, Lanzhou 730000, China; fyli@lzu.edu.cn (F.L.); puqs@lzu.edu.cn (Q.P.); zhanghx@lzu.edu.cn (H.Z.); zhanghuige@lzu.edu.cn (H.Z.); 2 CAS Key Laboratory of Special Pathogens and Biosafety, Center for Biosafety Mega-Science, Wuhan Institute of Virology, Chinese Academy of Sciences, Wuhan 430071, China; peace192@163.com (P.H.); xiongdongyan18@mails.ucas.ac.cn (D.X.); 3 College of Life Science, University of Chinese Academy of Sciences, Beijing 100049, China; 4 Zhengzhou Zhongdao Biotechnology Co., Ltd., Zhengzhou 450000, China; zblouyakun@126.com

**Keywords:** SARS-CoV-2, variant, VOC, RT-RAA

## Abstract

The worldwide pandemic caused by the severe acute respiratory syndrome coronavirus-2 (SARS-CoV-2) and its emergence of variants needs rapid and point-of-care testing methods for a broad diagnosis. The regular RT-qPCR is time-consuming and limited in central laboratories, so a broad and large-scale screening requirement calls for rapid and in situ methods. In this regard, a reverse transcription recombinase-aided amplification (RT-RAA) is proposed here for the rapid and point-of-care detection of SARS-CoV-2. A set of highly conserved primers and probes targeting more than 98% of SARS-CoV-2 strains, including currently circulating variants (four variants of concerns (VOCs) and three variants of interest (VOIs)), was used in this study. With the preferred primers, the RT-RAA assay showed a 100% specificity to SARS-CoV-2 from eight other respiratory RNA viruses. Moreover, the assay here is of a high sensitivity and 0.48 copies/μL can be detected within 25 min at a constant temperature (42 °C), which can be realized on portable equipment. Furthermore, the RT-RAA assay demonstrated its high agreement for the detection of SARS-CoV-2 in clinical specimens compared with RT-qPCR. The rapid, simple and point-of-care RT-RAA method is expected to be an appealing detection tool to detect SARS-CoV-2, including variants, in clinical diagnostic applications.

## 1. Introduction

The severe acute respiratory syndrome coronavirus-2 (SARS-CoV-2) has caused the worldwide COVID-19 pandemic [1,2] and has led to more than 216 million cases and 4.5 million deaths globally as of 1 September 2021, according to the real-time report of the COVID-19 epidemic from WHO [3]. It has brought huge economic and social burden all over the world [4]. SARS-CoV-2 undergoes inherent gene mutation during the worldwide spread [5] and the global pandemic promotes the emergence of variants [6]. In particular, the rapid emergence of variants of concerns (VOCs), including B.1.351 [7], P.1 [8], B.1.1.7 [9] and B.1.617.2 [10], and variants of interest (VOIs) [11], has attracted persistent public attention due to their unusual divergence and potential impact on biological importance [12,13]. The variation resulting from virus adaptive evolution endows the virus with the ability to escape the detection of the developed PCR methods by the current primer/probe sets if mutations happen within the primers and the probes in term of diagnosis, thus resulting in false negative results [9,14,15].

PCR methods based on highly conserved primer/probe sets targeting more SARS-CoV-2 strains are essential for the accurate detection of viral infections. Reverse transcription polymerase chain reaction (RT-PCR) technology has been widely accepted as the standard criteria in the diagnosis of the infectious diseases and has played a key role in SARS-CoV-2 detection [16]. However, its limitations are obvious, including having no capability in medical-resource-limited areas due to its requirement of well-trained technicians and expensive laboratory instruments, and the fact that it’s time-consuming and labor-intensive [17]. Therefore, there is an urgent need for developing a simple, rapid and point-of-care (POC) detection for SARS-CoV-2 to deal with the greatly rapid transmission of the virus and provide timely therapeutic schedules [18]. 

Isothermal nucleic acid amplification assays have no need for a thermal cycler, thus simplifying the operation process, and therefore attracting great attention in recent years to develop rapid and POC detection methods as alternatives of regular PCR. Among these isothermal assays, the recombinase-aided amplification (RAA) is a remarkably appealing isothermal amplification technique that reacts at a much lower temperature (37–42 °C) and in a much short time (usually within 20 min). The amplification utilizes recombinase derived from bacteria or fungi and single-strand DNA binding protein in order to realize room temperature amplification [19,20]. RAA has been studied to detect pathogens [21,22,23,24], including SARS-CoV-2 [19,20,25,26]. However, the feasibility of those methods toward SARS-CoV-2 variants, including currently circulating VOCs, has not been discussed yet. It is essential to develop a RAA test with highly conserved primer/probe sets in order to cover as many SARS-CoV-2 variants as possible in large-scale pathogen screenings and cope with the imminent danger worldwide due to the variants’ spread.

In this study, we developed a fluorescent-probe-based real-time reverse transcription-RAA (RT-RAA) assay. Primer/probe sets targeting a highly conserved 172 bp sequence located within orf1ab of SARS-CoV-2 were designed, and they covered 98% of SARS-CoV-2 strains, including the currently circulating variants (four VOCs and three VOIs), according to the analysis by an in-house program. Then, an optimal primer/probe set was obtained after screening. With the preferred primer/probe set, the RAA method showed a high specificity to SARS-CoV-2 compared to eight other respiratory RNA viruses, and a good sensitivity with the limit of detection of 0.48 copies/μL. Furthermore, its clinical application was demonstrated by 80 clinical specimens in comparison with the standard RT-qPCR. The RAA-based method for the detection of SARS-CoV-2 nucleic acid with a high sensitivity is potentially applicable for point-of-care testing (POCT) screenings of the infections of the pathogen. 

## 2. Materials and Methods

### 2.1. Reagents and Equipment

The QIAamp Viral RNA mini Kit (Qiagen, Hilden, Germany) was used for the virus RNA genome extraction. Takara one step PrimeScript RT PCR kit (RR064A, Takara, Japan) was used for the real-time amplification of RNA. The RT-RAA basic fluorescent kit (Zhengzhou Zhongdao Bio-Tech Co. Ltd., Zhengzhou, China) was used for RT-RAA assay of RNA. DEPC water (DNase, RNase free) (Beyotime Biotechnology, Shanghai, China) was used to dilute and treat RNA-involved samples. The primers and the probe (Table 1) were synthesized by Sangon Bio-Tech Co. Ltd., Shanghai, China. The coverage of the primers and probe was checked by an in-house program. Ultrapure water (18.2 MΩ·cm) was used in the study. All reagents were used as received, without further purification. 

C1000 thermal cycler (CFX96, Bio-rad, Hercules, CA, USA) was used for the real-time RT-PCR assay, and fluorescence detector (ZD-1610, Zhengzhou Zhongdao Bio-Tech Co. Ltd., Zhengzhou, China) was used for RT-RAA assay. 

### 2.2. Clinical Specimen Collection

A total of 80 throat swab specimens were collected from the suspected SARS-CoV-2 infectious patients based on the clinical indications and the CDC guidance from The First People’s Hospital of Jiangxia District, Wuhan city, China, from 25 January to 6 February 2020.

### 2.3. RNA Extraction

The clinical specimens were firstly treated in 60 °C for 1 h to inactivate the virus. Then, the virus RNA genome was extracted using the QIAamp Viral RNA mini Kit according to the manufacturer’s instructions. The finally obtained RNA samples were dissolved in DNase and RNase free water, aliquoted in 100 μL and stored at −80 °C until use.

### 2.4. Primer and Probe Design and Coverage Determination

Primers and a probe were designed according to the published sequence of orf1ab of SARS-CoV-2 on NCBI blast bank (GenBank Accession No. NC_045512.2, Position: 11553–11724). The software Primer 5.0 was used to select and analyze the primers and the probe according to the principles of RAA primer and probe design. The coverage of the primers and probe was checked by an in-house program. The coverage rate was determined by dividing the number of isolates with the same sequences of the primer/probe sequences by the total number of isolates until 6 March 2021 (a total of around 360,000 sequences with high quality). All of the primers and the probe were synthesized by Sangon.

### 2.5. RT-qPCR Assay

The RT-qPCR assays were performed in a reaction volume of 25 μL using a one-step RT-qPCR kit. The primer sequences for RBD of SARS-CoV-2 were listed as follows, unless otherwise stated: forward (Primer F) 5′-CAATGGTTTAACAGGCACAGG-3′; reverse (Primer R) 5′-CTCAAGTGTCTGTGGATCACG-3′; probe 5′-ACAGCATCAGTAGTGTCAGCAATGTCTC-3′, with the fluorophore of FAM and the quencher of BHQ1 at 5′ and 3′ end, respectively. The mixture for each reaction contained 12.5 μL of 2 × buffer, 4.9 μL of DNase-free water, 5 μL of extracted RNA template, 1 μL of primer F (10 μM), 1 μL of primer R (10 μM) and 0.6 μL of the probe (10 μM). The tubes were mixed upside down and shortly centrifuged to collect all of the solution down to the bottom of the tubes. The tubes were then placed into the C1000 thermal cycler and the cycling was shown as follows: heating at 42 °C for 5 min, then heating at 95 °C for 10 s and finally 40 cycles of denaturation at 95 °C for 5 s and annealing/extension at 60 °C for 30 s. 

### 2.6. RT-RAA Assay

The RT-RAA assays were performed in a reaction volume of 50 μL using a RT-RAA kit. The reaction mixture contained 5 μL of extracted RNA template, 29.4 μL of reaction buffer, the total volume of RNase-free water, primer F (10 μM), primer R (10 μM) and the probe (10 μM), which was 13.1 μL. Finally, 2.5 μL of 280 mM magnesium acetate was added in the lid of the tubes. The tubes were mixed upside down immediately and shortly centrifuged to collect all the solution down to the bottom of the tubes. Afterward, the tubes were placed into the fluorescence detector and measured for 25 min at 42 °C with the fluorescent signal collection at every 30 s.

### 2.7. Specificity Evaluation of RT-RAA Assay

Eight respiratory RNA viruses, including influenza A viruses (H3N2, H7N9, H5N1, H1N1) and influenza B viruses (Victoria and Yamagata lineages), which were isolated from human or birds and adenoviruses (AdV3 of strain IVCAS16(A).00027 and AdV7 of strain IVCAS 16(A).00028) were used to assess their RNA genome amplification by the RT-RAA assay to determine the specificity of the method. The RNA genome extraction and RT-RAA processes were the same as described above. The concentrations of the eight respiratory RNA genomes of the viruses were determined by RT-qPCR assay.

### 2.8. Clinical Specimen Identification

Both RT-qPCR and RT-RAA assays for detecting SARS-CoV-2 in 80 clinical throat-swab samples were performed following the methods of the RT-qPCR Assay and RT-RAA Assay sections described above, respectively. The results of the RT-RAA assay were compared to those of RT-qPCR assay to evaluate the real application of RT-RAA assay.

## 3. Results

### 3.1. Primer Screening

**Figure 1:**
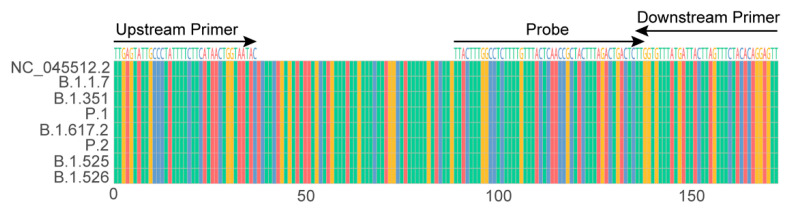
Sequence alignment of 172 targeted sequence among SARS-CoV-2 strain NC_045512.2 (GenBank Accession No.), four VOCs (B.1.1.7, B.1.351, P.1 and B.1.617.2) and three VOIs (P.2, B.1.525 and B.1.526).

The orf1ab sequence of the SARS-CoV-2 viral genome is one of the most frequently used targets in RT-qPCR detection for the virus identification. A 172 bp sequence located within the orf1ab sequence was selected from strain NC_045512.2 (GenBank Accession No.), which is the reported genome sequence in the early stage of the pandemic [27]. The coverage of the sequences of the upstream primers, downstream primers and the probe was checked as 98% of approximately 360,000 genome sequences of SARS-CoV-2 with a high quality by an in-house program; moreover, the 172 sequence was also highly conserved in the currently circulating variants (four VOCs and three VOIs) (Figure 1), indicating that the primers/probe candidates were highly conserved and had the ability to detect variants, including these VOCs and VOIs. 

According to the guidance of the RAA primer design, both the length and sequence greatly influence the amplification efficiency of the RAA assay, so primer screening is essential to reveal a suitable primer pair for a sensitive and rapid RAA assay. Eleven upstream primers and 10 downstream primers, with their 3′ end truncated one nucleotide gradually, were designed as the candidates to be screened, and a fluorescent probe was also designed to couple with the primers to conduct a fluorescent RAA assay (Table 1).

**Figure 2:**
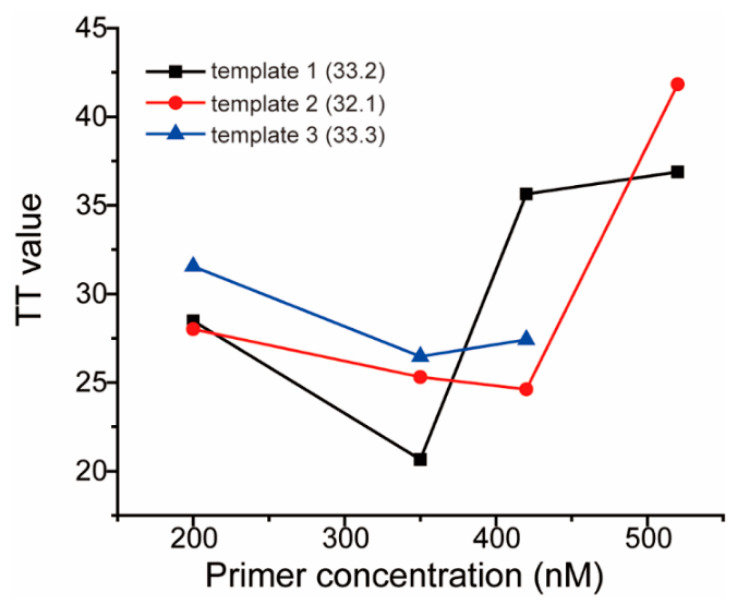
Optimizing the concentrations of the primer pair.

Before primer screening, optimization for primer concentration was performed to find a suitable primer amount in the RAA system. Randomly, a primer pair of F7/R9 was used and four concentrations were assessed. Three clinical specimen RNA were adjusted to similar concentrations as the templates through pre-quantifying by RT-qPCR. In the RAA assay, the TT value, which is the threshold time when the fluorescent signal in the tube reaches the preset threshold value, which is similar to the cycle threshold (*Ct)* value of qPCR, is related to the concentration of the RNA in the sample. As a result, 350 nM of primers showed lower TT values for all three templates (Figure 2), so this parameter was used in the following tests.

**Figure 3:**
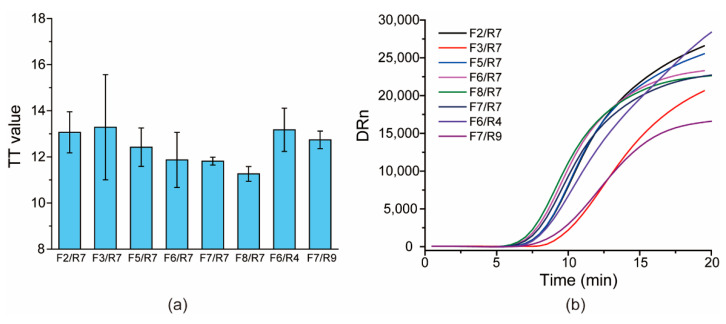
Screening primers: (**a**) the histogram of TT values versus different primer pairs; (**b**) the typical amplification curves for each primer pair.

For the primer screening, all of the upstream and downstream primers were paired in an orthogonal format to assess their amplification performance. Through comparison, eight pairs of the primer candidates were first selected due to their relatively lower TT values for templates derived from one clinical specimen diluted in RNase-free water (Figure 3), which has shown that three primer pairs (F7/R7, F8/R7, F7/R9) have a relatively low TT and good repeatability (small error bars for the three pairs). Then, the three primer pairs were applied to test several clinical RNA samples, and the TT of the F7/R9 pair was the lowest for all tested clinical specimens (data not shown); therefore, the pair F7/R9 was selected as the preferred primer pair and used in the following assay.

The target sequence refers to the orf1ab gene from the SARS-CoV-2 isolate Wuhan-Hu-1 (NCBI Reference Sequence: NC_045512.2 Position: 11553–11724)

### 3.2. Evaluation of the Specificity of the RAA System

**Figure 4:**
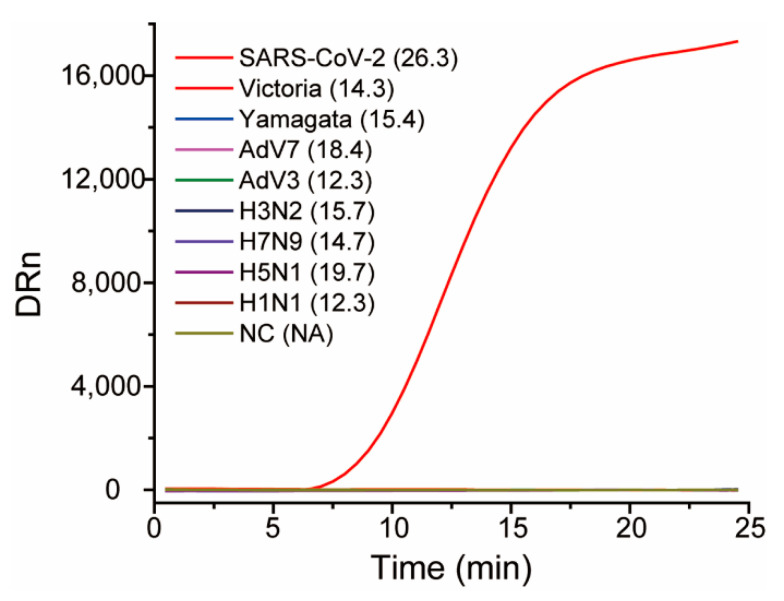
The typical amplification curves for specificity evaluation. The numbers in the brackets following the names of the viruses are the *Ct* values of RT-qPCR using the primer/probe sets targeting the corresponding viruses, respectively.

Eight respiratory RNA viruses that can cause similar respiratory symptoms as SARS-CoV-2 were used as interferents to evaluate the specificity of the proposed RT-RAA assay to its target SARS-CoV-2. All of the non-targeted viral RNA genomes were extracted, and the corresponding concentrations of the viral RNA genomes were in the *Ct* range 12.3–19.7 by RT-qPCR using primer/probe sets of the corresponding viral nucleic acids, whereas the *Ct* value for the SARS-CoV-2 RNA genome was 26.3, indicating that the amount of eight non-targeted RNA genomes was far larger than that of SARS-CoV-2. As shown in Figure 4, all of the non-targeted RNA genomes generated similar baselines as the negative control (water), whereas SARS-CoV-2 showed a typical and significant fluorescent amplification curve (TT value: 12.7), demonstrating that the RT-RAA assay was specific to the target SARS-CoV-2.

### 3.3. Analytical Sensitivity

**Figure 5:**
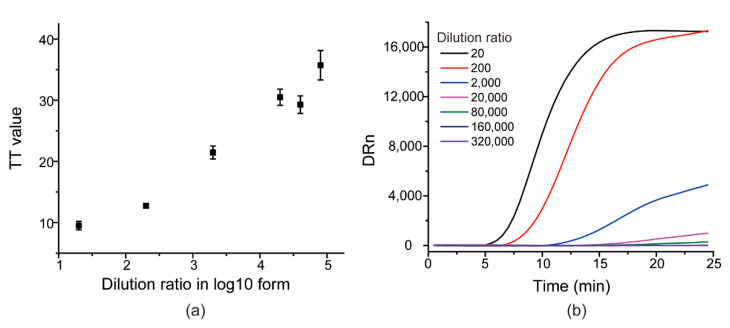
The scatter diagram of TT values versus dilution ratio of SARS-CoV-2 RNA genomes in log10 form (**a**) and the typical amplification curves of the seven templates (**b**).

The sensitivity of the RT-RAA assay for SARS-CoV-2 was evaluated by testing a set of serially diluted samples from 200× to 320,000× folds derived from one clinical specimen sample. All of the templates were pre-quantified by RT-qPCR. Each sample was tested thrice. As shown in Figure 5, the TT values were nearly linearly correlated to the dilution ratio of templates in log10 form, and the 8000× dilution produced an average TT value of 35.7 ± 2.4, whereas the other two higher dilutions 160,000× and 320,000× showed no significant peaks within 25 min, indicating that 160,000× has been undetected by RT-RAA, and that the lowest detected limit was the 8000× dilution by the proposed RT-RAA. For RT-qPCR, the *Ct* value for the 8000× dilution was 36.3 ± 1.3 and the calculated LOD was 0.48 copies/μL, according to the regression equation of the logarithm of RNA concentrations (copies/mL) versus *Ct* value obtained by the reverse transcription droplet digital polymerase chain reaction (RT-ddPCR) [28].

### 3.4. Clinical Specimen Detection

To evaluate the clinical application of the RT-RAA assays in terms of SARS-CoV-2 detection, 80 clinical specimens were tested. RT-qPCR was also carried out as the reference in order to validate the amplification performance of the RT-RAA assay. As shown in Table 2, for the 80 clinical specimens, RT-qPCR identified 69 positives (*Ct* value ranging from 22.1 to 36.4) and 11 negatives (one with *Ct* > 37.0 and ten with *Ct* > 40.0). For the 69 positives, 45 (*Ct* value ranging from 22.1 to 32.8) showed 44 positives by the proposed RT-RAA assay, showing a sensitivity of 98%, whereas the remaining 24 (*Ct* value ranging from 33.2 to 36.4) only showed eight positive and 16 negatives, giving a sensitivity of 33%, indicating that RAA displayed a high agreement when *Ct* was lower than 33, but gave precarious results when the template was low with a *Ct* value higher than 33. For the 11 negatives, all were negative results using the RT-RAA test, providing a specificity of 100% (Table 2). 

These results showed the high consistency of RT-RAA with RT-qPCR for *Ct* less than 33, and the lower sensitivity of RT-RAA for *Ct* higher than 33. RAA is expected to be advantageous as a rapid and in situ method in the broad screening of pathogens outside well-equipped areas.

## 4. Discussion

The epidemic of SARS-CoV-2 around the world has posed a profound impact on the life and economy of all of the involved countries. Its adaptive evolution results in genetic variations that have been promoted by the global pandemic, giving huge challenges to the diagnosis and vaccine development. Variation offers the virus the ability to escape detection when mutated sites are located in primers and the probe in the developed molecular diagnosis method, leading to the ineffectiveness of the methods in detecting the variants. With the emergence of SARS-CoV-2 variants, especially several circulating VOCs and VOIs, it is important to find and use highly conserved primers for any nucleic acid detection method in order to cover as many variants as possible. 

Diverse nucleic acid detection methods with conserved sequences, including orf1ab, N, S and E genes as the targets and their corresponding primer/probe sets, have demonstrated their vital roles in the battle with SARS-CoV-2, but their performances toward the variants were rarely discussed. A further and persistent effort toward developing more rapid and effective detection methods using highly conserved primers/probe sets is significant and worthwhile. 

Among diverse molecular diagnosis methods, RAA has aroused great public interest in recent years due to its unique features, including reacting at a low and constant temperature, being thermal-cycler-free and having several downstream detection formats. RAA can be potentially prominent for a broad and large-scale pathogen screening as an economical POC tool in medical-resource-limited settings. Compared to other POC methods based on the molecular diagnosis and serological diagnosis toward SARS-CoV-2 [29,30], the uniqueness of RAA is obvious in its effective amplification, simple operation and low cost.

A highly conserved sequence located within the orf1ab was selected here to conduct an RAA assay to identify SARS-CoV-2 in clinical throat swab specimens. Its primers and probe can detect 98% of SARS-CoV-2 strains through an analysis of 360,000 sequences according to our analysis; more importantly, they covered currently circulating variants, including the four most-discussed VOCs B.1.351, P.1, B.1.617.2 and B.1.1.7, indicating that the proposed RAA assay was able to detect SARS-CoV-2 variants, including the four VOCs. Through the orthogonal combination of the upstream and downstream primers, a pair showed the lowest TT values for clinical specimens, and thus were selected as the optimal pair. Together with the probe aligning the conserved sequence, the proposed RT-RAA assay displayed a high sensitivity to SARS-CoV-2 and no cross-reactivity was observed with eight respiratory RNA viruses. 

The results here validated the effectivity and usefulness of the RT-RAA assay in SARS-CoV-2 detection. With the primer pair and probe, the RAA assay achieved a sensitivity of 0.48 copies/μL, which was at a similar level to the previous reported 1 copy/μL [25], 1 copy/reaction and 10 copies/reaction [19,20], suggesting that the selected primers and probe were reasonable. In terms of a real application, 80 clinical specimens were collected and tested by RT-RAA, and the agreement in virus infection identification was 98% when compared with the standard RT-qPCR when *Ct* was lower than 33 (*n* = 45), whereas it was 33% when *Ct* was higher than 33 (*n* = 24). This reveals that RAA is still not as sensitive as PCR, especially for complex samples. The extract from real clinical specimens seems to influence RAA amplification negatively; this is probably because the much lower and constant reaction temperature (42 °C) makes RAA amplification more sensitive to interference derived from small amounts of residual components after the viral genome is separated from clinical specimens, whereas PCR is more robust due to its steps at a much higher temperature (95 °C). Despite this, the worth of RAA as a pre-detection tool for SARS-CoV-2 in a broad and large-scale screening is still significant, particularly in medical-resource-limited settings. 

In conclusion, a rapid, simple and in situ RT-RAA assay targeting SARS-CoV-2 with 98% gene coverage of around 360,000 sequences with a high quality until 6 March 2021 was proposed, and its effectiveness in SARS-CoV-2 detection was validated. The portability and simplicity of the equipment makes the RAA assay an appealing POC detection tool in medical-resource-limited settings for a broad and large-scale SARS-CoV-2 screening. 

## Figures and Tables

**Table 1:** The sequences of primers and probe used in this study.

Primers/Probe	Sequences (5′-3′)
Upstream	F1: TTGAGTATTGCCCTATTTTCTTCATAACTGGTAATAC F2: TTGAGTATTGCCCTATTTTCTTCATAACTGGTAATA F3: TTGAGTATTGCCCTATTTTCTTCATAACTGGTAAT F4: TTGAGTATTGCCCTATTTTCTTCATAACTGGTAA F5: TTGAGTATTGCCCTATTTTCTTCATAACTGGTA F6: TTGAGTATTGCCCTATTTTCTTCATAACTGGT F7: TTGAGTATTGCCCTATTTTCTTCATAACTGG F8: TTGAGTATTGCCCTATTTTCTTCATAACTG F9: TTGAGTATTGCCCTATTTTCTTCATAACT F10: TTGAGTATTGCCCTATTTTCTTCATAAC F11: TTGAGTATTGCCCTATTTTCTTCATAA
Downstream	R1: AACTCCTGTGTAGAAACTAAGTAATCATAAACACCA R2: AACTCCTGTGTAGAAACTAAGTAATCATAAACACC R3: AACTCCTGTGTAGAAACTAAGTAATCATAAACAC R4: AACTCCTGTGTAGAAACTAAGTAATCATAAACA R5: AACTCCTGTGTAGAAACTAAGTAATCATAAAC R6: AACTCCTGTGTAGAAACTAAGTAATCATAAA R7: AACTCCTGTGTAGAAACTAAGTAATCATAA R8: AACTCCTGTGTAGAAACTAAGTAATCATA R9: AACTCCTGTGTAGAAACTAAGTAATCAT R10: AACTCCTGTGTAGAAACTAAGTAATCA
Probe	TTACTTTGGCCTCTTTTGTTTACTCAACCGC[FAM-dT]A[THF] [BHQ1-dT]TTAGACTGACTCTTG[spacer C3]

**Note**: FAM, 6-carboxyfluorescein; THF, tetrahydrofuran; BHQ: black hole quencher; spacer C3: 3′-phosphate blocker.

**Table 2:** Comparison between RT-RAA and RT-qPCR for detection of clinical specimen.

	RT-qPCR (*Ct*)	RT-RAA	Sensitivity (%)	Specificity (%)
Positive	45 (22.1–32.8)	44	98 ^1^	
	24 (33.2–36.4)	8	33 ^1^	
Negative	11 (>37.0)	11		100 ^2^

^1^ 44/45 × 100% = 98% (*Ct* < 33). 8/24 × 100% = 33% (*Ct* > 33). ^2^ 11/11 × 100% = 100%.

## Data Availability

All available data are presented in this manuscript.
